# Cell-Free DNA, High-Mobility Group Box-1, and Procalcitonin Concentrations in Dogs With Gastric Dilatation–Volvulus Syndrome

**DOI:** 10.3389/fvets.2018.00067

**Published:** 2018-04-09

**Authors:** Roberta Troia, Massimo Giunti, Stefano Calipa, Robert Goggs

**Affiliations:** ^1^Department of Veterinary Medical Sciences, Alma Mater Studiorum, University of Bologna, Bologna, Italy; ^2^Department of Clinical Sciences, College of Veterinary Medicine, Cornell University, Ithaca, NY, United States

**Keywords:** cell-free DNA, procalcitonin, high-mobility group box-1, biomarker, gastric necrosis, ischemia–reperfusion injury, prognosis

## Abstract

Canine gastric dilatation–volvulus (GDV) is a life-threatening disease characterized by extensive tissue ischemia, tissue hypoperfusion, and systemic inflammation. Biomarkers that better reflect the severity of gastric necrosis and systemic inflammation would aid clinicians in the management of these patients. This study aimed to investigate the prognostic significance of cell-free DNA (cfDNA), high-mobility group box-1 (HMGB1), and procalcitonin (PCT) in dogs with GDV. Concentrations of cfDNA, HMGB1, and PCT were measured in citrated plasma samples collected from 29 dogs with GDV at hospital admission. Additional data collected included baseline lactate concentrations, APPLE_fast_ score, evidence of gastric necrosis, occurrence of postoperative complications, and outcome. Twenty-four healthy dogs were sampled as controls. Continuous variables between groups were compared with the Mann–Whitney *U* and correlations between continuous variables were assessed by calculation of Spearman’s correlation coefficient. Alpha was set at 0.05. Dogs with GDV had significantly greater concentrations of cfDNA, HMGB1, and PCT compared to controls (*P* = 0.0009, *P* = 0.004, and *P* = 0.009, respectively). PCT concentrations were significantly higher in non-survivors compared to survivors (*P* = 0.008). Dogs with gastric necrosis had significantly greater lactate concentrations compared to dogs without gastric necrosis (*P* = 0.0005). The APPLE_fast_ score was not prognostic. Lactate and PCT concentrations were moderately, positively correlated (*r*_s_ 0.51, *P* = 0.0005). Concentrations of the inflammatory biomarkers cfDNA, HMGB1, and PCT are increased in canine GDV. Only lactate and PCT concentrations were prognostic in this population of GDV dogs and were predictive of the presence of gastric necrosis and of non-survival to hospital discharge, respectively.

## Introduction

Canine gastric dilatation–volvulus (GDV) syndrome is a life-threatening emergency, characterized by pain, abdominal distension, systemic inflammation, and severe tissue hypoperfusion. The pathophysiology of the disease involves rapid intragastric gas accumulation, volvulus, and progressive increases in intragastric pressure. Increased tissue pressure causes ischemia with local cell injury and death that can progress to tissue necrosis. Hypovolemia from third-space fluid loss, coupled with obstructive shock due to decreased venous return rapidly follow, leading to secondary organ dysfunction (1). The prognosis for GDV is favorable with early diagnosis, timely medical stabilization, and prompt surgical correction (2). Systemic complications can develop during the course of the syndrome or in the early postoperative period, however, including sepsis and reperfusion injury that can lead to multiple organ dysfunction and death (1, 2). The presence and the extent of gastric necrosis are reported as a strong predictor of mortality in GDV dogs (1).

Dogs with GDV frequently fulfill the criteria for the presence of systemic inflammatory response syndrome (SIRS) (3). Tissue hypoperfusion, cellular necrosis, and ischemia-reperfusion injury are the main triggers of SIRS in the perioperative period (3, 4), and increased plasma concentrations of SIRS-associated biomarkers, such as C-reactive protein and high-mobility group box-1 (HMGB1) have been reported in GDV dogs (4, 5). Blood lactate concentrations have been demonstrated to be prognostic in GDV syndrome (1). Serial lactate monitoring has been advocated in the postoperative period to identify surgical dehiscence (1) and decreased lactate clearance has been associated with gastric necrosis and mortality (6, 7). The prevalence of hyperlactatemia at presentation is variable in dogs with GDV, however, and baseline lactate concentrations are not consistently prognostic (5–7). Hence, there is a need for novel biomarkers that are prognostic early in the course of GDV syndrome, and that parallel the extent of tissue ischemia and systemic inflammation.

Plasma cell-free DNA (cfDNA) has been receiving growing attention as a biomarker in critically ill humans. The precise origin of this circulating cfDNA remains unclear, but cells undergoing necrosis or apoptosis in addition to leukocytes forming extracellular traps are the most likely sources (8, 9). In healthy people and in healthy dogs, plasma cfDNA concentrations are low, while increased concentrations have been detected in many conditions characterized by systemic inflammation and extensive tissue injury (10). Independent of the underlying disease, cfDNA concentrations reflect the severity of cell damage and are prognostic in cardiac arrest, ischemia/reperfusion injury, shock, trauma, and sepsis (9–12). The veterinary literature in this area is limited, but increased blood cfDNA concentrations have been documented in dogs with immune-mediated hemolytic anemia, cancer, sepsis, and trauma (13–15). A positive correlation between cfDNA concentration and disease severity has also been documented in a heterogeneous group of clinically ill dogs (16).

High-mobility group box-1 is a non-histone chromosomal protein that is highly conserved among species. In humans, HMGB1 is released passively after cellular necrosis (but not apoptosis), and is actively released from inflammatory cells as a late-phase cytokine (17). In the intravascular space, HMGB1 acts as an alarmin—a damage-associated molecular pattern that serves to enhance inflammation. HMGB1 plays a significant role in the course of ischemia–reperfusion injury and vasculitis (17, 18), and increased concentrations of HMGB1 have been documented in sick dogs (19–22). Concentrations of HMGB1 are of prognostic value in dogs with lymphoma, SIRS, and GDV (4, 19, 23).

Procalcitonin (PCT), the prohormone of calcitonin, has emerged as a diagnostic and prognostic biomarker in human sepsis. Concentrations of PCT can differentiate sepsis from non-infectious SIRS, be used to monitor response to therapy and to predict sepsis-related complications and prognosis (24, 25). The utility of circulating plasma PCT in dogs has been investigated in several studies. Baseline PCT concentration predicted organ dysfunction and serial PCT concentrations were related to outcome in canine sepsis (26). However, PCT was not able to differentiate dogs with sepsis from dogs with non-septic SIRS or non-septic SIRS from healthy control dogs (27). Although previous studies proposed PCT as a novel acute phase protein (APP) (28), its clinical value has not been extensively evaluated in dogs with systemic inflammation.

Thus, the aim of the current study is to assess the prognostic value of cfDNA, HMGB1, and PCT in dogs with GDV. We hypothesized that (1) concentrations of cfDNA, HMGB1, and PCT are increased at the time of presentation in dogs with GDV compared to healthy controls; (2) these biomarkers predict occurrence of gastric necrosis, postoperative complications, and survival to discharge; (3) concentrations of these biomarkers are positively correlated with each other, with lactate concentration and with an established illness severity score.

## Materials and Methods

### Animals

Canine plasma samples were collected from dogs managed at the two participating veterinary teaching hospitals (Cornell University, Ithaca, NY, USA and University of Bologna, Italy). Specifically, stored citrated plasma samples collected from dogs with GDV (Cornell *n* = 21, Bologna *n* = 8) enrolled as part of other studies were analyzed. Those studies were approved by the local Institutional Animal Care and Use Committees (IACUC), and undertaken under written informed client consent (Cornell IACUC 2014-0053; Bologna DL 26/2014, Project 581).

Inclusion criteria for this study included a diagnosis of GDV, and availability of an aliquot of citrated plasma collected at the time of admission and stored frozen at −80°C. The diagnosis of GDV syndrome was based on compatible clinical signs and the presence of characteristic gas distension and displacement of the stomach on right lateral abdominal radiographs (29). Respective primary clinicians determined all aspects of case management. Dogs euthanized at the time of presentation were excluded.

Twenty-four healthy privately owned dogs were enrolled as controls (Cornell IACUC 2014-0052; Bologna project ID 581). These dogs were eligible for inclusion if they had no history or evidence of recent or chronic medical conditions and had not received any medication, except for routine preventative health care, within the preceding 3 months. Dogs were classified healthy on the basis of history, physical examinations, and complete blood count and serum chemistry results.

### Clinical and Clinicopathologic Data Collection

From the dogs with GDV, the following data were recorded: physical examination findings at presentation, the presence or absence of gastric necrosis intraoperatively, the occurrence and nature of postoperative complications. Outcome was defined as survival to hospital discharge, death, or euthanasia. In presence of operable gastric necrosis, a partial gastrectomy was performed. If gastric necrosis was deemed inoperable according to the attending surgeon, dogs underwent intraoperative euthanasia. Blood was collected at the time of presentation and prior to patient stabilization for baseline laboratory analyses, including measurement of lactate concentrations (Lactate Pro 2, Arkray, Minneapolis, MN, USA; Lactate Scout Analyzer, Senslab, Leipzig, Germany). Blood collected into citrated plasma tubes containing 3.2% sodium citrate (1:9 ratio) were centrifuged (3,000 *g* for 10 min) immediately after collection, the supernatant decanted, and stored frozen at −80°C until analysis. Citrated plasma samples used in this study were collected between July 2015 and March 2017, with analyses performed in June 2017. Patient clinical and clinicopathologic data were used to evaluate the Acute Patient Physiologic and Laboratory Evaluation (APPLE_fast_) score as previously reported (30).

### cfDNA, HMGB-1, and PCT Evaluation

All biomarkers were measured as a batch at Cornell University. Samples from Bologna were transported to Cornell on dry ice by overnight courier. All samples were frozen on arrival and were stored at −80°C prior to analysis. All three analytes are reportedly stable for prolonged periods at −80°C (31–33). Citrated plasma samples were thawed at 37°C just prior to analysis. Measurement of cfDNA was performed with a fluorescence-based method using DNA-specific fluorophores (Quant-iT High Sensitivity DNA assay Kit, Life Technologies, Grand Island, NY, USA) as previously reported in dogs (14). Reagents were used per manufacturer’s instruction. Canine HMGB1 was quantified by means of a commercially available human sandwich ELISA kit (IBL-International, Hamburg, Germany) previously validated for use in dogs (20). PCT concentrations were measured using a commercial canine ELISA kit (Biovendor LLC, Asheville, NC, USA). Absorbance values for the ELISA assays were measured using a microplate reader (BioTek Instruments, Winooski, VT, USA). To improve within-run precision, concentrations of cfDNA were measured in triplicate. Owing to cost constraints, concentrations of HMGB1 and PCT were measured in duplicate only. All assays have coefficients of variation ≤10%. The mean values of replicates were used for subsequent analyses.

### Statistical Analysis

Prior to test selection, data were assessed for normality by assessment of histograms, calculation of skewness and kurtosis and with the D’Agostino Pearson test. Descriptive statistics were calculated as appropriate. Most data were nonparametric. Data are, therefore, presented as median (min–max). The Mann–Whitney *U*-test was used to compare continuous variables between groups. Spearman’s correlation coefficient was used to assess correlations between variables. Alpha was set at 0.05. All analyses were performed using commercially available software (Prism 6.0, GraphPad Software, La Jolla, CA, USA).

## Results

### Demographics

Twenty-nine dogs with GDV were enrolled. The study population included 12 castrated male dogs, 8 intact male dogs, 7 spayed female dogs, and 2 intact female dogs. The median age was 9 years (0.8–14). The median bodyweight was 37 kg (17.0–72.3). The median rectal temperature on presentation was 38.3°C (36.5–40.5), the median heart rate was 160 bpm (80–220), the median respiratory rate was 48 rpm (24–120). The median APPLE_fast_ score was 23 (10–40) and the median duration of hospital stay was 2 days (0–5). Of the 29 dogs, 6 were seen by a primary care veterinarian prior to assessment at the study institutions. All six were radiographed. Only three dogs received treatment prior to presentation at the study centers including fluid therapy (*n* = 2) and opioid analgesia (*n* = 2). Twenty-two dogs (76%) were discharged from the hospital and were considered survivors, while seven dogs (24%) did not survive to hospital discharge. All non-survivors were humanely euthanized. No dog was euthanized due to financial constraints. Evidence of gastric necrosis was detected in seven dogs during surgery: four dogs were euthanized intraoperatively for inoperable necrosis; 1 underwent partial gastrectomy but was euthanized later in the course of hospital stay for severe complications; 2 underwent partial gastrectomy and survived to hospital discharge. Twenty-two dogs had no evidence of gastric necrosis at the time of surgery. Of these, dogs 20 survived, while 2 were euthanized during the course of hospital stay for late complications. Systemic complications were reported in four dogs, and included pneumonia (*n* = 2), septic peritonitis (*n* = 1), and disseminated intravascular coagulation (*n* = 1). Twenty-four dogs were enrolled as controls. There were 13 spayed female dogs, 6 castrated male dogs, 3 intact female dogs, and 2 intact male dogs. The median age of the controls was 4 years (1–13), and the median body weight was 26 kg (5–62). The median age and body weight of GDV dogs were significantly higher compared to control dogs (*P* = 0.0002 and *P* = 0.010, respectively).

### cfDNA, HMGB1, and PCT Comparisons

**Figure 1:**
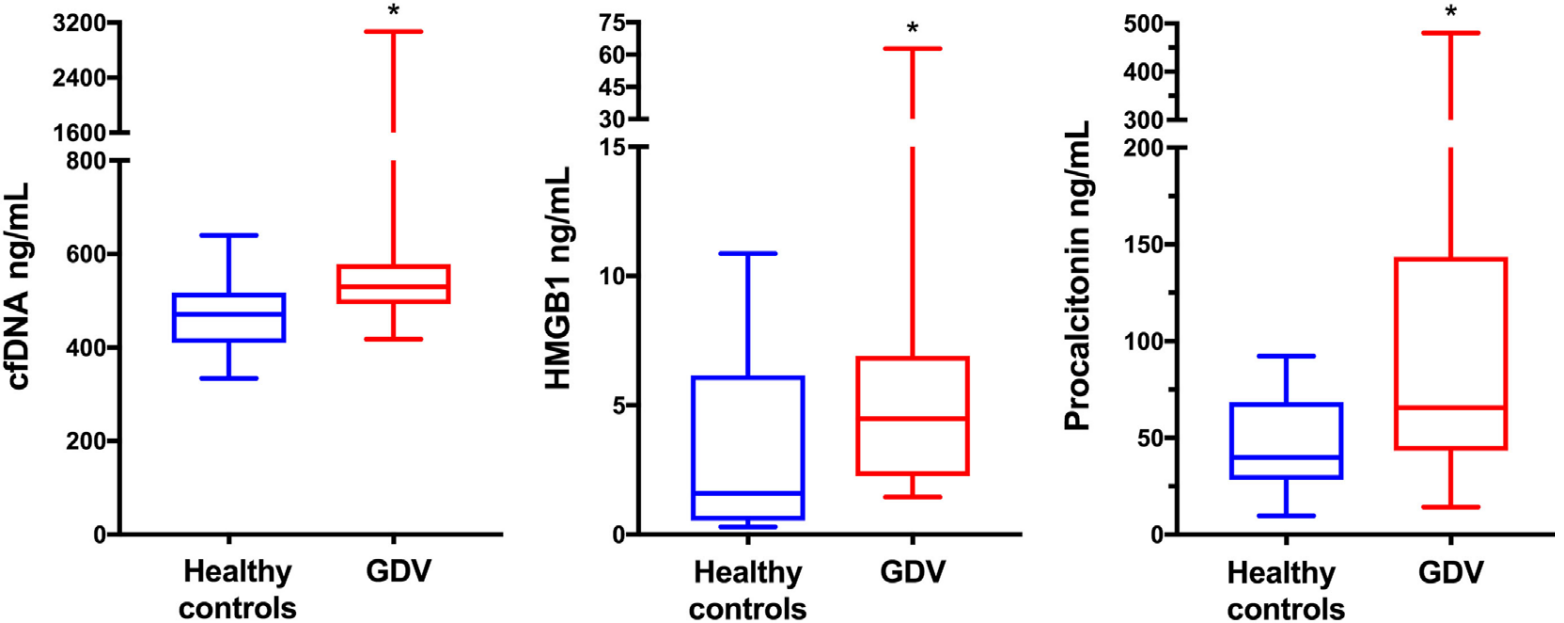
Box and whisker plot comparisons of plasma concentrations of cell-free DNA (cfDNA), high-mobility group box-1 (HMGB1), and procalcitonin (PCT) from 24 healthy control dogs and 29 dogs with gastric dilatation–volvulus (GDV) syndrome. The central line represents the median, the boxes represent the interquartile range and the whiskers represent the minimum and maximum values. * indicates statistical significance at *P* < 0.05. Dogs with GDV had significantly greater plasma concentrations of cfDNA, HMGB1 (*n* = 28) and PCT (*n* = 28) compared to healthy controls by Mann–Whitney *U*-test (*P* = 0.0009, *P* = 0.004, and *P* = 0.009, respectively).

**Figure 2:**
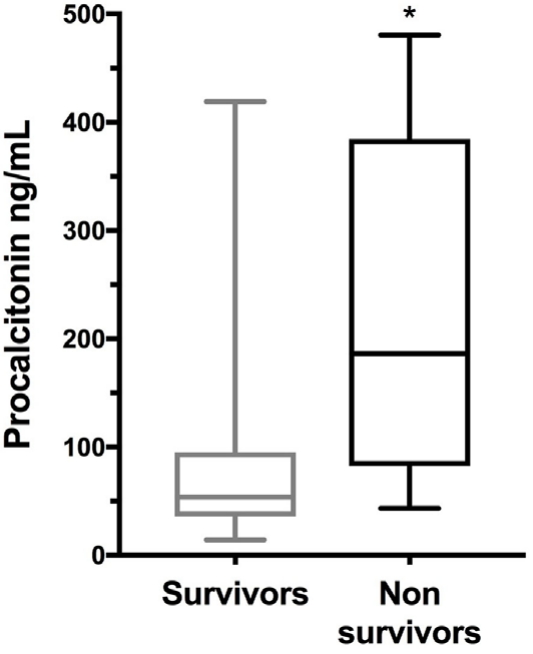
Box and whisker plot comparison of plasma procalcitonin (PCT) concentrations in 28 dogs with gastric dilatation–volvulus syndrome categorized as survivors (*n* = 21) and non-survivors (*n* = 7). The central line represents the median, the boxes represent the interquartile range, and the whiskers represent the minimum and maximum values. * indicates statistical significance at *P* < 0.05. Non-survivors had significantly greater plasma concentrations of PCT compared to survivors by Mann–Whitney *U*-test (*P* = 0.008).

The median concentrations of cfDNA (*n* = 29), HMGB1 (*n* = 28), and PCT (*n* = 28) were significantly greater in dogs with GDV compared to controls (*P* = 0.0009, *P* = 0.004, and *P* = 0.009, respectively) (Table 1; Figure 1). There was no difference between the cfDNA or HMGB1 concentrations of survivors and non-survivors (*P* = 0.35 and *P* = 0.32, respectively), but median PCT concentrations were significantly greater in non-survivors compared to survivors (*P* = 0.008) (Table 2; Figure 2). The difference in PCT concentrations between survivors and non-survivors remained significant even after the exclusion of the three dogs developing septic complications. There was no difference between the cfDNA, HMGB1, or PCT concentrations of dogs with or without gastric necrosis (*P* = 0.67, *P* = 0.26, and *P* = 0.66, respectively), or between dogs developing postsurgical complications compared to dogs that did not develop complications (*P* = 0.69, *P* = 0.59, and *P* = 0.06, respectively) (Table 2).

**Table 1:** Concentrations of measured inflammatory biomarkers in dogs with GDV compared to healthy controls.

Biomarker	Control dogs (*n* = 24)	GDV dogs (*n* = 29)	*P*
Cell-free DNA (ng/mL)	471 (334–640)	530 (418–3,070)	**0.0009**
HMGB1 (ng/mL)^a^	1.6 (0.3–10.9)	4.5 (1.5–62.8)	**0.004**
Procalcitonin (ng/mL)^a^	39.8 (9.7–92.3)	65.5 (14.2–480.6)	**0.009**

*Data are presented as median (min–max). *P*-values in bold type were significant at *P* < 0.05*.

*^a^GDV dogs n = 28*.

*GDV, gastric dilatation–volvulus; HMGB1, high-mobility group box-1*.

**Table 2:** Comparison of the variables under analysis in GDV dogs according to final outcome and presence of gastric necrosis.

Biomarker	Survivors (*n* = 22)	Non-survivors (*n* = 7)	*P*
Cell-free DNA (cfDNA) (ng/mL)	523 (418–677)	534 (484–3,070)	0.35
HMGB1 (ng/mL)^a^	4.5 (1.5–13.7)	5.9 (1.5–62.8)	0.32
Procalcitonin (PCT) (ng/mL)^a^	53.7 (14.2–419.2)	186.4 (43.3–480.6)	**0.008**
Lactate (mmol/L)	2.8 (1.1–10.6)	5.2 (2.6–11.9)	0.05
APPLE_fast_ score	23 (12–40)	22 (10–35)	0.76

**Biomarker**	**Gastric necrosis absent (*n* = 22)**	**Gastric necrosis present (*n* = 7)**	***P***

cfDNA (ng/mL)	523 (418–677)	547 (446–3,070)	0.43
HMGB1 (ng/mL)^b^	4.5 (1.5–13.8)	5.9 (1.6–62.8)	0.60
PCT (ng/mL)^b^	61.5 (20.5–419.2)	82.5 (14.2–480.6)	0.57
Lactate (mmol/L)	2.5 (1.1–10.6)	9.7 (4.9–11.9)	**0.0005**
APPLE_fast_ score	23 (12–40)	25 (19–35)	0.17

*Data are presented as median (min–max). *P*-values in bold type were significant at *P* < 0.05*.

*^a^n = 21 survivors*.

*^b^n = 21 gastric necrosis absent*.

*GDV, gastric dilatation–volvulus; HMGB1, high-mobility group box-1*.

### Additional Results

**Figure 3:**
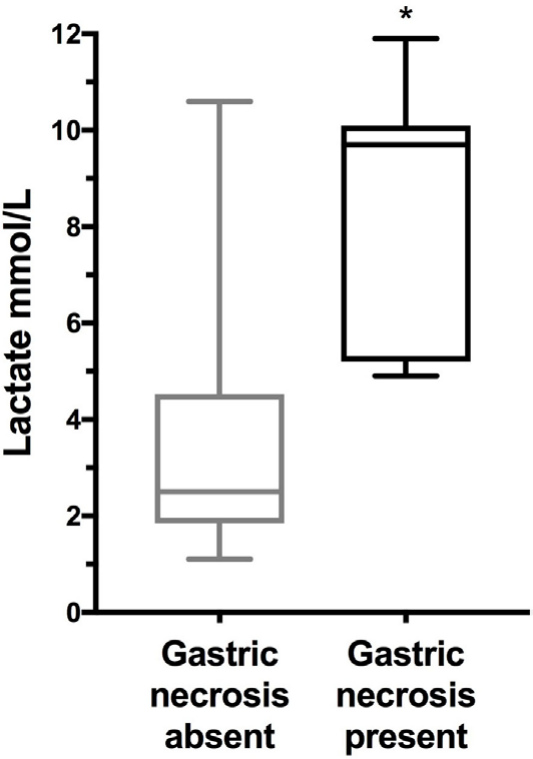
Box and whisker plot comparison of blood lactate concentrations from 22 dogs with gastric dilatation–volvulus (GDV) without gastric necrosis, and 7 dogs with GDV and gastric necrosis. The central line represents the median, the boxes represent the interquartile range, and the whiskers represent the minimum and maximum values. * indicates statistical significance at *P* < 0.05. Dogs with gastric necrosis had significantly higher lactate concentrations compared to dogs without gastric necrosis by Mann–Whitney *U*-test (*P* = 0.0005).

**Figure 4:**
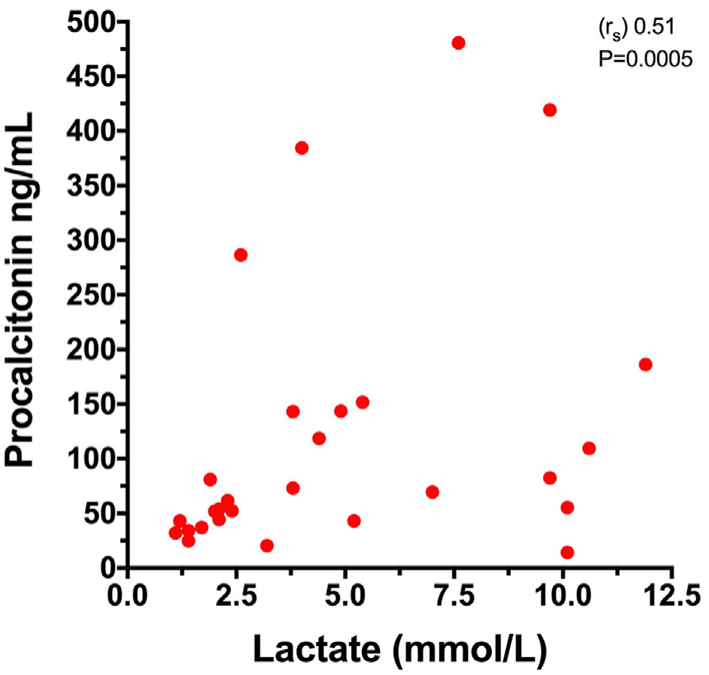
Comparison of plasma procalcitonin and blood lactate concentrations in 28 dogs with gastric dilatation–volvulus (GDV) syndrome. Spearman’s rank correlation coefficient (*r*_s_) 0.51, *P* = 0.0005.

Mortality rate was significantly higher in dogs with gastric necrosis compared to dogs without gastric necrosis 5/7 (71.4%) vs. 2/22 (9.1%) (*P* = 0.003). The median blood lactate concentration was significantly higher in dogs with gastric necrosis compared to dogs without gastric necrosis (*P* = 0.0007) (Table 2; Figure 3), but lactate concentration was not different between survivors and non-survivors (*P* = 0.053), or between dogs with versus without postsurgical complications (*P* = 0.30). The APPLE_fast_ score was not different between survivors and non-survivors (*P* = 0.76), between dogs with versus without gastric necrosis (*P* = 0.17), or between dogs with or without postsurgical complications (*P* = 0.96). Blood lactate and plasma PCT concentrations were moderately and significantly positively correlated (*r*_s_ 0.51, *P* = 0.0005) (Figure 4), but no other significant correlations were identified between any of the biomarkers themselves or between blood lactate concentrations and APPLE_fast_ score.

## Discussion

The present study investigated the prognostic ability of novel biomarkers of cellular damage and inflammation in dogs with GDV. Consistent with our hypothesis and in agreement with the existing literature, dogs with GDV syndrome had significantly greater median plasma cfDNA, HMGB1, and PCT concentrations compared to healthy controls. Human and canine data suggests that cfDNA in the plasma derives from neutrophil extracellular trap formation and cell death (9, 10, 14). As such, the greater concentrations of cfDNA documented in GDV dogs could originate from gastric ischemia and necrosis, or from tissue damage resulting from global hypoperfusion and systemic inflammation. Similar mechanisms likely explain the increased HMGB1 concentrations documented in our GDV dogs compared to healthy dogs, as HMGB1 can be passively released by necrotic cells (17). Interestingly, however, concentrations of cfDNA and HMGB1 were not significantly correlated, which points to different sources of these two biomarkers. Given that it is a late-phase cytokine, the increases in HMGB1 concentration noted may indicate SIRS rather than local gastric tissue injury (19).

Increased transcription of PCT mRNA has been reported in canine SIRS due to infectious and non-infectious causes (28, 34), and a resultant increase in circulating PCT protein has been documented in septic dogs (26, 27). The causes of the increased circulating PCT concentrations in dogs with GDV are, as yet, unknown. PCT may act as an APP, as previously suggested (28), or it may be associated with septic phenomena during GDV syndrome, since bacterial translocation has been reported in dogs with GDV (35), and septic complications (e.g., septic peritonitis) are commonly documented perioperatively (2, 3).

It was hypothesized that cfDNA and HMGB1 reflect the degree of cell damage and, therefore, would predict gastric necrosis and outcome in canine GDV. Data from the present study do not support this hypothesis, however, because neither of these biomarkers correlated with gastric necrosis or were prognostic in this population. It is possible that the cfDNA concentrations are not predictive because they represent a single snapshot of cell damage/necrosis at the time of presentation. The processes causing gastric necrosis continue until the point of surgical correction and hence cfDNA concentration might escalate from the time of presentation to the time of surgery. Obtaining these samples would be more logistically difficult, but might be more discriminant than observation at presentation. As a parallel, although cfDNA concentrations are prognostic in humans with bacteremia (36, 37), they are not prognostic in cardiac arrest, a syndrome characterized by ischemia and reperfusion injury (9). Additionally, mechanisms other than necrosis have been postulated to explain the increase in plasma concentrations of cfDNA in people, including neutrophil extracellular trap formation and the active release of cfDNA as a method of intercellular communication (8, 10).

Uhrikova et al. reported HMGB1 to be prognostic in dogs with GDV, wherein higher baseline and postsurgery concentrations were documented in non-survivors (4). Those authors hypothesized that given the peracute nature of GDV syndrome and the kinetics of HMGB1 release, the increase in HMGB1 reflected gastric necrosis rather than systemic hypoperfusion and inflammation (4). The median HMGB1 concentrations measured in GDV dogs in the present study (in both survivors and non-survivors) are comparable with the values reported for survivors only in the study by Uhrikova et al. This suggests that the dogs in the present study may have been less affected overall than those previously reported. Additional dissimilarities between the two study populations, such as time from onset of clinical signs to presentation, and potential variations in assay conduct may account for the divergent results. Three dogs in the present study received treatment prior to study enrollment. Given the small number of dogs that received therapy prior to blood sampling, it is unlikely that this impacted our findings; however, we cannot completely exclude an effect on biomarker concentrations. None of the dogs that received prior treatment died or had gastric necrosis.

Surprisingly, no correlation was documented between cfDNA and HMGB1. Although plasma concentrations of both these biomarkers indicate the release of cellular nucleic acids into circulation, their increase might be regulated by different pathways with distinct kinetics. To date, only weak correlations have been reported between cfDNA and DNA-histone-complexes in people after cardiac arrest (9), and between cfDNA and HMGB1 in dogs with sepsis (38). The lack of correlation between the two biomarkers and lactate concentrations might also reflect different stimuli for the release of these markers.

Procalcitonin measured in samples collected at the time of presentation was the only variable able to predict outcome in this population of GDV dogs, although it should be noted that there was considerable overlap in the PCT concentrations between these two groups. PCT is a promising biomarker to aid in sepsis diagnosis and prognostication in people (39–41), and there is limited but growing evidence that PCT might also be a useful biomarker in dogs (28, 34). An earlier effort to measure PCT in dogs was unsuccessful, owing to problems with the ELISA (42), but the present study used a different assay from the Floras et al. study. PCT clearance is prognostic in dogs with sepsis, and baseline PCT was associated with the occurrence of organ dysfunction and septic shock (26). Although the prognostic value of baseline PCT remains unclear in people and dogs with sepsis (27, 43), its usefulness in the course of canine GDV syndrome may result from the typically short duration of this disease. The difference in PCT concentrations between survivors and non-survivors remained significant even after the exclusion of the three dogs developing septic complications, suggesting a utility of PCT in GDV dogs beyond an association with the development of sepsis. Previous studies have not documented a relationship between C-reactive protein and outcome in dogs with GDV (4, 5). A comparison between PCT and other more well-established canine APPs was beyond the scope of the present study, but would be of interest. Further investigation will be necessary to determine whether PCT behaves as an APP during GDV, if it mirrors SIRS severity, or if it relates to the development of late organ dysfunction.

The moderate positive correlation identified between plasma PCT and blood lactate concentrations in the present study is consistent with the relationships between these biomarkers and the development of complications in these patients. The present study again serves to confirm the clinical value of lactate measurements in dogs with GDV, since this was the only biomarker that was related to the presence of gastric necrosis. It should be noted, however, that no cut-off value that was 100% sensitive and specific for gastric necrosis, owing to the degree of overlap between the two groups. Lactate measurement is inexpensive and easy to perform at the point of care. Although PCT measurement appeared to offer useful information in these dogs, there is currently no commercial point-of-care test for canine PCT, limiting the ease of measurement. Development of such an assay might enhance the ability of clinicians to manage dogs with GDV, however.

The present study has some limitations. A convenience sampling method was used, which limited the number of samples available for analysis. This may have reduced the power of some of our analyses. The identification and the assessment of the severity of gastric necrosis was subjective. Some dogs were euthanized based on these assessments and as such, it cannot be determined what the outcome would have been in those patients had surgery been completed and the dogs allowed to recover from anesthesia. Although blood sample collection was performed prospectively, medical records were retrospectively reviewed. In some cases, some data were missing. For example, time from onset of clinical signs to presentation, or time from presentation to the start of surgery were not universally determined. As such, the relationship between these factors could not be assessed and their potential impact on the biomarkers concentrations is unknown. Finally, minor differences in the management of the enrolled GDV dogs (stabilization measures, anesthetic protocols, surgical procedures, and postoperative care) between the two teaching hospitals involved in the study may have influenced the outcomes and introduced variability.

In conclusion, concentrations of cfDNA and HMGB1 are increased in dogs with GDV, potentially due to cellular damage, systemic inflammation, and tissue hypoperfusion. Neither cfDNA or HMGB1 concentrations were prognostic in the present population of dogs with GDV. In contrast, plasma PCT concentrations were predictive of outcome and were moderately, positively correlated with lactate concentrations. Blood lactate concentrations were the only biomarker to correlate with the presence of gastric necrosis. The combination of lactate and PCT concentrations might provide valuable guidance for clinicians managing dogs with GDV.

## Ethics Statement

All samples analyzed in this study were collected from dogs managed at the two participating veterinary teaching hospitals (Cornell University, Ithaca, NY, USA and University of Bologna, Italy) as part of studies approved by the local Institutional Animal Care and Use Committees (IACUC), and undertaken under written informed client consent (Cornell IACUC 2014-0053; Bologna DL 26/2014, Project 581). Healthy privately owned dogs were enrolled as controls with local Institutional Animal Care and Use Committees approval and written informed client consent (Cornell IACUC 2014-0052; Bologna project ID 581).

## Author Contributions

RT assisted with study design, collected and analyzed data, and co-wrote the manuscript; SC and MG assisted with study design, collected and analyzed data, and edited the manuscript; RG designed the study, analyzed data, and co-wrote the manuscript. All authors contributed to, read, and approved the final manuscript.

## Conflict of Interest Statement

The authors declare that the research was conducted in the absence of any commercial or financial relationships that could be construed as a potential conflict of interest.

## Abbreviations

APP, acute phase protein; APPLE, acute patient physiologic and laboratory evaluation; cfDNA, cell-free DNA; GDV, gastric dilatation–volvulus; HMGB1, high-mobility group box-1; PCT, procalcitonin; SIRS, systemic inflammatory response syndrome.
